# Human rights dimensions of food, health and care in children’s homes in Kampala, Uganda – a qualitative study

**DOI:** 10.1186/s12914-016-0086-y

**Published:** 2016-03-18

**Authors:** Line Erikstad Vogt, Byaruhanga Rukooko, Per Ole Iversen, Wenche Barth Eide

**Affiliations:** Department of Nutrition, Institute of Basic Medical Sciences, University of Oslo, Oslo, Norway; School of Liberal and Performing Arts, Makerere University, Kampala, Uganda; Division of Human Nutrition, Faculty of Health Sciences, Stellenbosch University, Tygerberg, South Africa

**Keywords:** Adolescent girls, Care, Children’s homes, Child care institutions, Human rights, Nutrition, Right to food

## Abstract

**Background:**

More than 14 % of Ugandan children are orphaned and many live in children’s homes. Ugandan authorities have targeted adolescent girls as a priority group for nutrition interventions as safeguarding nutritional health before pregnancy can reduce the chance of passing on malnutrition to the offspring and thus future generations. Ugandan authorities have obligations under international human rights law to progressively realise the rights to adequate food, health and care for all Ugandan children. Two objectives guided this study in children’s homes: (a) To examine female adolescent residents’ experiences, attitudes and views regarding: (i) eating patterns and food, (ii) health conditions, and (iii) care practices; and (b) to consider if the conditions in the homes comply with human rights standards and principles for the promotion of the rights to adequate food, health and care.

**Methods:**

A human rights-based approach guided the planning and conduct of this study. Five children’s homes in Kampala were included where focus group discussions were held with girls aged 12-14 and 15-17 years. These discussions were analysed through a phenomenological approach. The conditions of food, health and care as experienced by the girls, were compared with international standards for the realisation of the human rights to adequate food, health and care.

**Results:**

Food, health and care conditions varied greatly across the five homes. In some of these the girls consumed only one meal per day and had no access to clean drinking water, soap, toilet paper and sanitary napkins. The realisation of the right to adequate food for the girls was not met in three homes, the realisation of the right to health was not met in two homes, and the realisation of the right to care was not met in one home.

**Conclusions:**

In three of the selected children’s homes human rights standards for food, health or care were not met. Care in the children’s homes was an important contributing factor for whether standards for the rights to adequate food and health were met.

## Background

### Malnutrition, orphans and children’s homes in Uganda

Despite being rich in natural resources and having favourable conditions for agriculture, malnutrition, poverty and disease impose serious economic and social constraints on Uganda [1–3]. Malnutrition in particular is regarded as one of the biggest challenges in achieving economic growth and human welfare [4]. The causes of malnutrition are complex and multifaceted, and many factors can contribute to a person becoming malnourished. Adolescent girls constitute a particularly vulnerable group in regard to nutrition, given that good nutritional status before and during pregnancy can prevent adverse transmission of risk of malnutrition to foetuses and further to future generations [5, 6]. Almost one out of four Ugandan girls (24 %) aged 15 – 19 years is already a mother or pregnant with their first child. They are also likely to be deficient in both micro- and micronutrients [7]. Notably, inadequate maternal nutritional status before conception, short maternal stature, and poor nutritional intake during pregnancy, are all associated with intrauterine growth retardation and can result in low birth weight infants.

Fourteen percent of all children in Uganda are orphaned, equating to about 2.43 million children. This is predominantly the result of high prevalence of HIV/AIDS, the high level of poverty leading to poor health, poor access to health facilities, inadequate hygiene and living conditions, diseases such as malaria and tuberculosis, poor social protection systems, gender inequality, a high fertility rate, a high level of inadequate parental care practices, and an overstretching of the Ugandan informal child protection structures [8–11].

The high number of orphans in Uganda correlates with the high number of children’s homes, about 600. An “approved home” is a governmental or non-governmental home approved by the Minister of Children’s Affairs to provide substitute family care for children below 18 years. The *Approved Homes Regulations* contain a few guidelines regarding care practices, and factors contributing to treatment and prevention of disease [12]. As of June 2012 the number of children living in child care institutions in Uganda was estimated to exceed 57 000 [13].

The Ministry of Gender, Labour and Social Development (MGLSD) conducted in 2011 a study on 40 children’s homes in Uganda. The findings revealed that many limitations characterize these homes: More than 65 % of them lacked qualified human resources, over 50 % had inadequate child care provisions, and only 20 % had a child protection policy. Furthermore, the MGLSD study revealed that there was insufficient access to clean drinking water, diet was very limited, and children were looking malnourished [13].

### Theoretical frameworks

This study focused on adolescent girls in selected orphanages in Uganda. Two theoretical frameworks were used to guide the study.

**Fig. 1:**
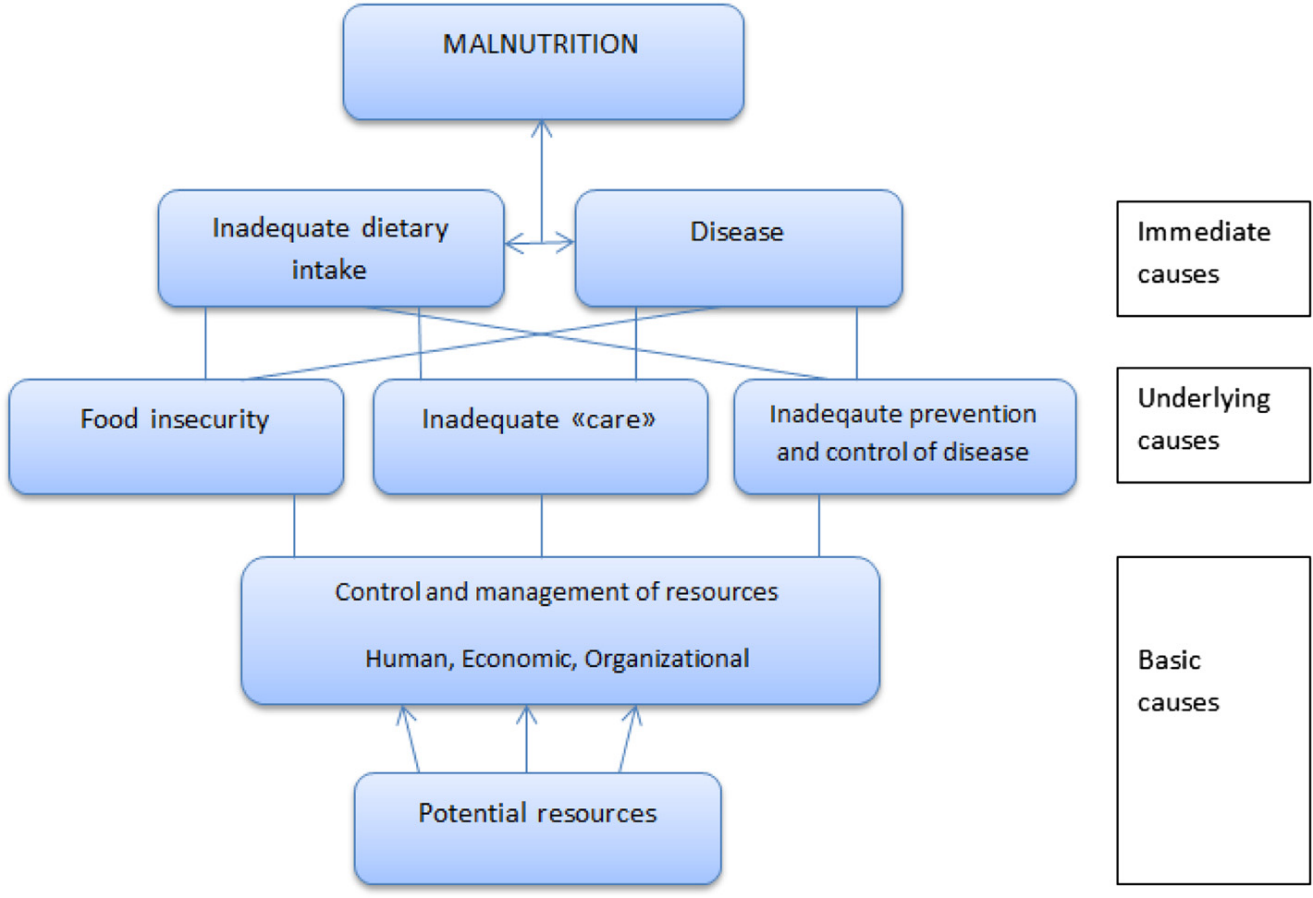
UNICEF’s Conceptual Framework for the cause of malnutrition. Adapted from UNICEF (1998)

**Fig. 2:**
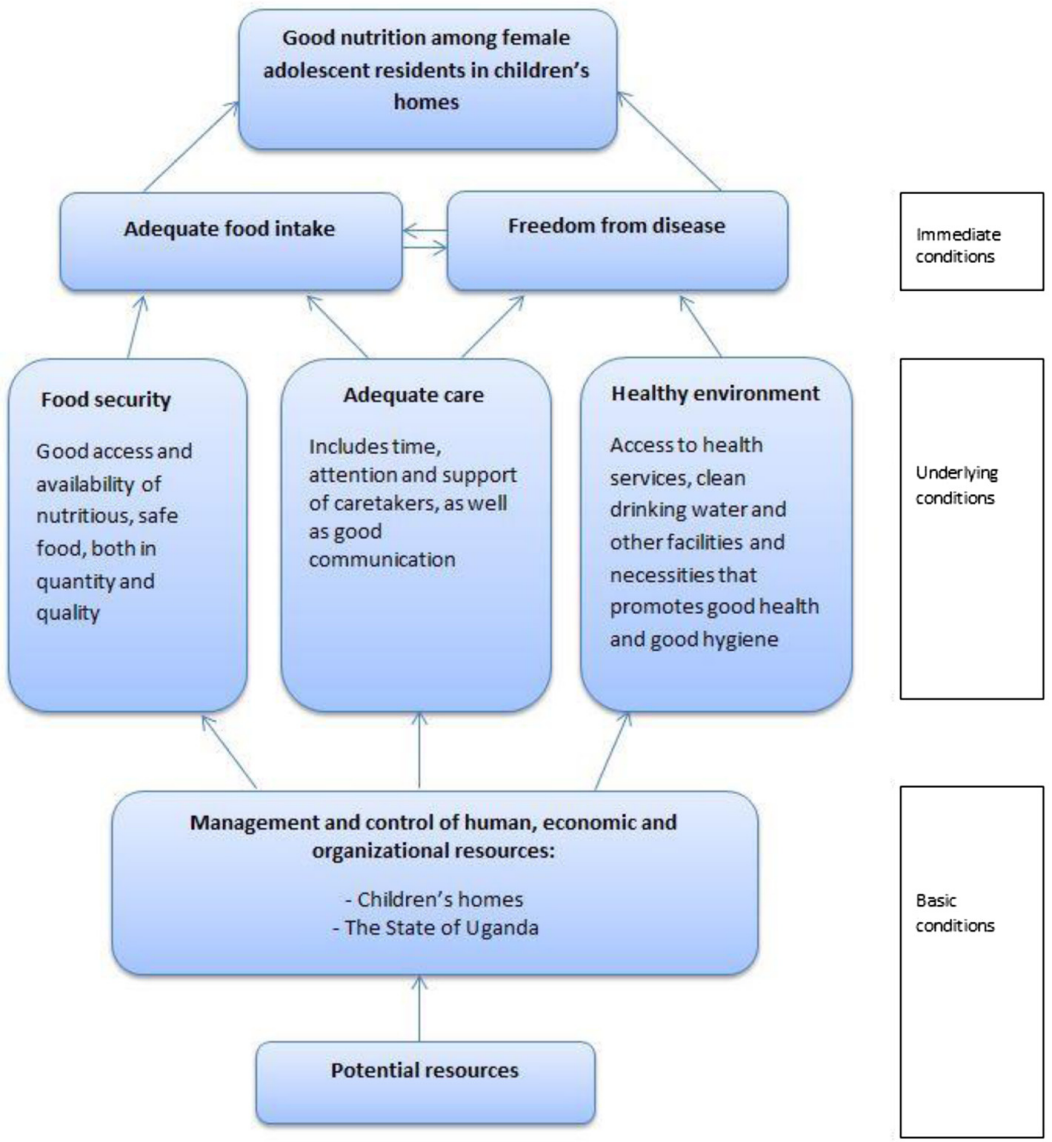
Normative conceptual framework for good nutrition among adolescent girls living in children’s homes. Adapted from Engesveen (2005)

Firstly, UNICEF’s conceptual framework (Fig. 1) is an analytic tool developed to portray causal determinants of malnutrition. The roots of child malnutrition are categorized into three levels; immediate, underlying and basic causes. One can also consider the reverse: the conditions necessary at the same levels for achieving good nutrition. The UNICEF conceptual framework is thereby “turned around” to become a normative framework that can help conceptualise what measures are necessary to ensure these conditions for good nutrition (Fig. 2). Adequate food intake and freedom of disease are the immediate conditions for good nutrition. The underlying conditions are clustered under food security, adequate care and a proper healthy environment, which also constitutes the necessary components of nutrition security as defined by WHO: “a situation in which food security is combined with a clean environment, adequate health services, and appropriate care and feeding practices, to ensure a healthy life for all household members” [14].

The concept “care” has been defined as “the provision in the household and the community of time, attention and support to meet the physical, mental and social needs of the growing child and other household members” [15]. “Care” also refers to aspects such as how food is distributed within the household, time of caretakers, emotional support, material support and the level of education, attitudes, skills and practices performed by caretakers to meet the physical, social and mental needs of children [16–19].

A “proper healthy environment” exists when all members at the household level have adequate access to basic health services and health care, and are living in an environment that promotes good health. This includes access to clean drinking water, sanitation and shelter. If adequate conditions for good health exist there is a decrease in morbidity which is one of the conditions for optimal nutrition [20].

Secondly, on the basis of Uganda’s national and international obligations to progressively realise the human rights to food, health and care for all Ugandan juveniles, as well as the country’s focused attention towards protecting rights of vulnerable children, we saw the need to examine the situation in children’s homes in more detail from a human rights perspective. Human rights are universal and apply to each individual and sometimes groups of individuals. Human rights establish legal entitlements for rights-holders and corresponding legal obligations for duty-bearers to uphold those entitlements. The human right to adequate food and to be free from hunger and the right to the highest attainable standard of health are recognised in the the International Covenant on Economic, Social and Cultural Rights (ICESCR) and the Convention on the Rights of the Child (CRC). The CRC also recognises the right to care [21, 22].

**Fig. 3:**
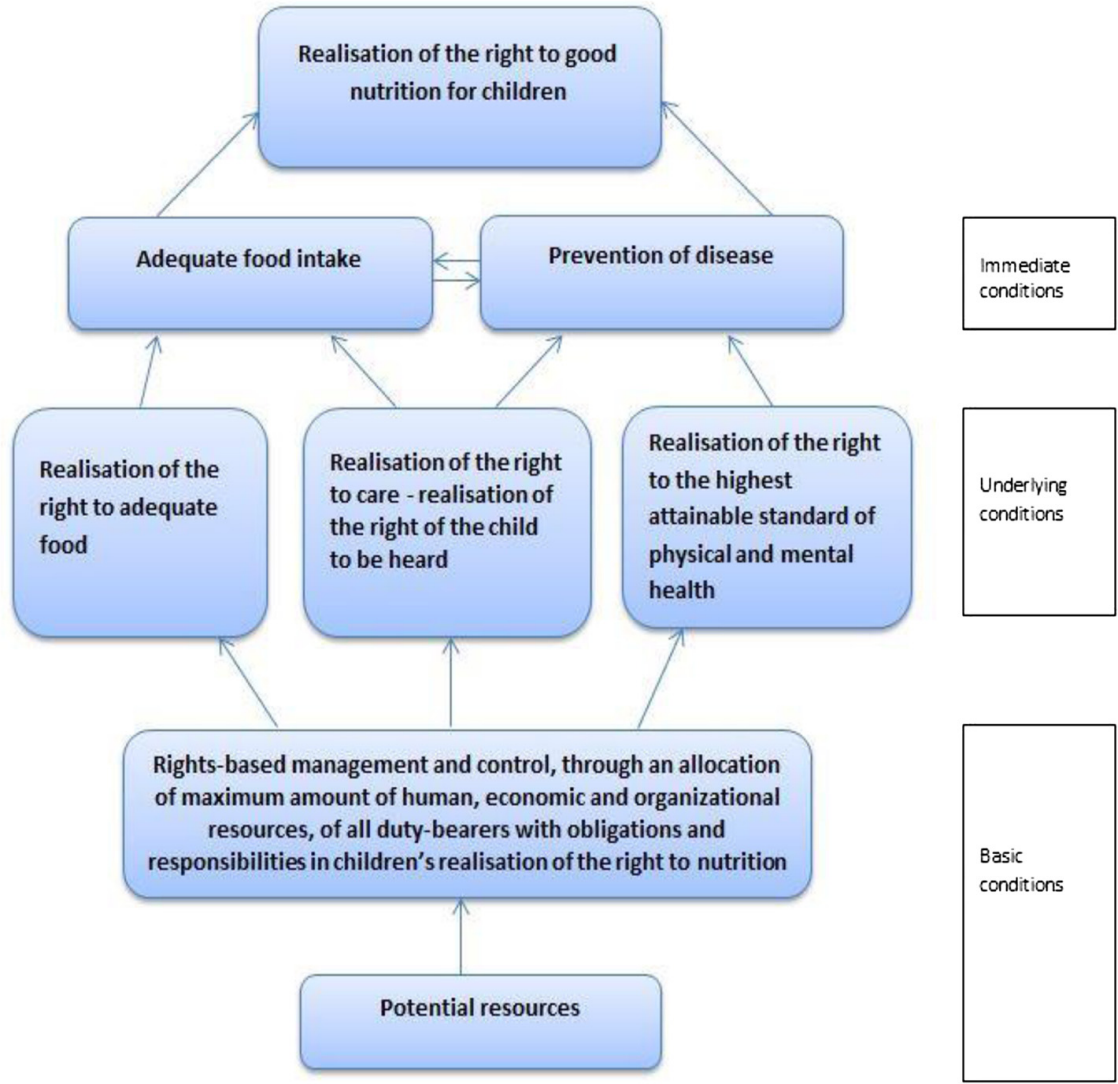
Adaption of the normative framework for good nutrition, where the ultimate goal is realisation of the right to good nutrition, and underlying conditions are substituted with human rights standards. The changes according to the basic conditions (from the framework for good nutrition) are that duty-bearers take a rights-based approach in all aspects of management and control under their job descriptions, including the way they allocate their potential resources. The underlying conditions are realisation of the right to adequate food, realisation of the right to care – where realisation of the right of the child to be heard is included (to be explained later in this chapter), and the realisation of the right to the highest attainable standard of health. Realisation of these rights will allow for an adequate food intake and prevention of disease of children within the resources available. While the normative framework for good nutrition recognises the immediate condition concerning health as “freedom from disease”, the right to the highest attainable standard of health does not equate to the right to be healthy. This current framework for the realisation of the right to good nutrition must be understood through the human rights language where children are entitled to access to healthcare and basic facilities and necessities that promotes good health and prevents disease, but where health and freedom of disease is not a human right itself

Seen together with the UNICEF conceptual framework, the necessary conditions for good nutrition can be taken a step further and seen as the realisation of these rights (Fig. 3). We sought to find out if the conditions in the children’s homes comply with standards and principles for the rights to adequate food, health and care, and to the right to freely express oneself and be heard. The opportunity to have one’s opinion heard and be taken seriously is one of the fundamental values of the CRC, and can also have a bearing on the enjoyment of other rights.

Our study applied a human rights perspective on data collection and in discussing the findings. The aim of the study was to investigate specific experiences of adolescent girls in selected children’s homes in Uganda’s capital Kampala regarding conditions that can affect their nutrition situation. They included (i) the adolescent girls’ experiences, attitudes and views regarding eating patterns and food as served; (ii) health conditions, including access to health care and other basic necessities that promote good health; and (iii) care practices as manifested through relationships and communication with caretakers in the children’s homes. The second complementary aim was to consider if the conditions in the homes complied with human rights standards and principles for the rights to adequate food, health and care.

## Methods

This study was planned and conducted within the frame of a human rights-based approach (HRBA). A central element of a HRBA is to focus on the most vulnerable groups in society. Adolescent female children’s homes residents constitute one such highly vulnerable group. The principles of a HRBA were developed by the United Nations to guide developmental policies or the conduct of interventions by applying certain human rights principles and standards relevant to the task [23]. No intervention was conducted in this study, and usage of a HRBA was related to applying human rights principles and standards in planning, data collection and analyses.

The human rights principles that guided this study were *non-discrimination, participation, empowerment,* and *human dignity* applied to the adolescents as rights-holders..We also considered certain aspects of two other principles as they related to performance of the children’s homes, *respect for the rule of law* and *accountability*.

Standards regarding the rights to food, health and care were drawn from the two legal instruments in question (ICESCR and CRC) and the authoritative interpretations by their the respective UN committees in the form of “General Comments” (GC), here as regards the meaning of the specific rights in question for this study – food, health and hygiene, and care.

### Selection of children’s homes

The data was collected in Kampala from February till May 2013. We included five, unisex children’s homes. For a children’s home to be selected a minimum of ten adolescent girls should have lived there ≥ three months. Consequently, among the 47 children’s homes in Kampala identified by Ugandan authorities, 20 homes were eligible, and five of these were randomly selected using an internet-based software (www.randomization.com). These five children’s homes will be referred to as CH A to E. Prior to data collection each of these homes were visited to inform the staff about the study, and written introductory letters was brought from the MGLSD and the University of Oslo. Prior to the commencement of the actual data collection, one children’s home in Kampala was assessed in a pilot study to familiarize the research team with the environment and to sharpen the research tools.

### Selection of study participants

The inclusion criteria for participation in this study were:

- Girls aged 12-17 years.
- Girls who had been living in the selected children’s home for a minimum of three months.
- Girls who were fluent in either English or Luganda (the local language in Kampala).

We applied “intercept recruiting”, which means that the participants are found at the location where the focus group will be held [24], in this case at the specific children’s home. We assembled all girls in the given age group to inform them about the study. The girls who first expressed that they wanted to participate were selected to take part in the study. All these girls were eligible according to the inclusion criteria and thus none were excluded from the study.

### Focus group discussions

A qualitative approach was adopted for this study, due to its applicability of capturing subjective meanings to a certain phenomenon, and understanding life according to the perspective of people who live it themselves [25–27].

It was decided to conduct two discussions at each home in order to get opinions from adolescent girls within different age groups, i.e. those aged 12 – 14 years (*n* = 22) and those aged 15 -17 years (*n* = 21). The number of participants in each focus group varied between four and five.

The focus group discussions were held outdoors in the late afternoon after the girls had come home from school. The recommendations from Malterud were followed [28]. At the beginning of each session, the participants were affirmed that their participation was anonymous, and that none of their answers could be attributed to them personally. The participants were also asked to respect each other’s privacy and anonymity by not sharing anyone’s stories outside the group. A recorder was used to capture the discussions on tape. Each group discussion lasted between 1¼ to 2 h.

A research assistant facilitated the focus group discussions. She was born and lived in Kampala and was fluent in both English and Luganda.

### Analyses of the focus group discussions

We neither analysed facial impressions nor non-verbal communication between the participants, but solely emphasized the verbal material of the participants for analysis.

All ten focus group discussions were transcribed *verbatim* from the tape recorder the day after the group interview had been conducted. The software program “ATLAS.ti” (ATLAS.ti Scientific Software Development GmbH, Berlin, Germany) was used for systematic organizations of the transcripts for further analysis.

Malterud’s systematic text condensation (STC), an adaptation of Giorgi’s phenomenological analysis [29–31], was used as guide for data analysis of the focus group discussions. STC is a descriptive approach. The researcher does not try to interpret possible underlying meanings of what the participants have communicated, but aims at presenting their experiences as expressed by the participants themselves. However, as the researcher is not a children’s home resident and can thereby only see the participants from an “outside perspective”, the analysis is based on the researcher’s interpretation and understanding of how the participants view and experience their own lives.

### Analysing data in light of human rights standards

After processing data from the focus group discussions through the STC, the participants’ explanation of their food, health and care situation was compared to human rights standards drawn from interpretations of the meaning of the specific right in the general comments to the human rights convention in question. The conditions of food, health and care, as experienced by the adolescent girls, were aligned with the following human rights standards as interpreted by the Committee on Economic, Social and Cultural Rights (CESCR), and the Committee on the Rights of the Child:

- GC no. 12 of the CESCR on the Right to Adequate Food: Article 11 of the International Covenant on Economic Social and Cultural Rights [32]
- GC no. 14 of the CESCR on the Right to the Highest Attainable Standard of Physical and Mental Health: Article 12 of the International Covenant on Economic Social and Cultural Rights [33]
- GC no. 12 of the Committee on the Right of the Child on the Right of the Child to be Heard: Article 12 of the Convention on the Rights of the Child [34]

The participants’ experiences of food and health conditions were considered in relation to interpretations in GC 12 on the right to adequate food and GC 14 on the right to the highest attainable standard of health, respectively. There is no general comment on the right of the child to care, although the CRC explicitly recognises the right to care in article 3(2). This study has therefore drawn connections to how GC 12 of the CRC on right of the child to be heard correlates with the definition of care as defined at the International Conference on Nutrition (ICN) in 1992 [15]. Perceptions of care by the participants were thus compared to both provisions in GC 12 on the right of the child to be heard, and to interpretations of how care as defined at the ICN in 1992 can be adapted to the children’s home context.

### Ethical considerations

Participation in the study regarding both the individual children’s home and the individual girl was voluntary, as according to the Helsinki Declaration. In line with this the study was approved by the Uganda National Council for Science and Technology, The Office of the President in Uganda (no. YCH/201/288/01), and the Norwegian Regional Committee for Medical and Health Research Ethics (no. 2013/578). The managers of each children’s home gave verbal permission to carry out focus group discussions with the girls, and a written informed consent was obtained from each girl participating in the study. As there was a possibility that not all the girls had effective literacy skills, the research assistant read and translated information and the informed consent to them. They thereby had adequate information to make a voluntary decision of whether to participate or not. This consent procedure was developed for the participating girls and it was approved by the relevant Ugandan and Norwegian committees. The focus group discussions were conducted with only the participants, a researcher and a research assistant present. It was always made sure that the discussions were held in a place where nobody else could hear the interaction. Hence, the participants did not risk to be victimized by their caretakers.

## Results

### Characteristics of study participants and children’s homes

All five children’s homes are privately operated and owned. None of them had MGLSD- approved home status at the time of data collection. All had one or more caretakers that were present at the home on a 24 h basis. All the residents slept in dormitories with bunk beds, however, the number of children at each dormitory varied across the homes. Everyone slept under mosquito nets to prevent malaria. Furthermore, all residents were HIV-tested when they joined the homes.

All school-aged children attended school, most of them from Monday to Friday. The homes paid for their tuition, school uniforms, books etc. None of the participating girls had part-time jobs or any means to receive money. Hence they were totally reliant on the children’s homes in terms of food, school fees, health services and basic necessities.

The most frequent dish served in all children’s homes in Uganda is posho and beans. Customarily, most primary and secondary schools in Uganda also serve posho and beans for lunch on a daily basis. According to all managers spoken to in this study, posho and beans are the cheapest foods one can get in Uganda. Posho is one of the many staple dishes and consists of highly refined white maize meal that is cooked with water until a solid consistency. Other dishes served in children’s homes were also in line with the traditional Ugandan cuisine, e.g. matoke, cassava, sweet potatoes, potatoes, rice, sorghum and millet. Meat and chicken were usually served on special occasions such as Christmas and Easter. The girls who received breakfast in the children’s homes ate maize porridge.

### Data from the focus group discussions

Food and eating patterns varied greatly across the five children’s homes in Kampala. Table 1 summarizes the meal frequency and type of food served across these homes. It differentiates between the youngest and oldest participants at each home, as their food intake often differed according to whether they were enrolled in primary or secondary school.

**Table 1:** Main trends in meal frequency and food served across the five children’s homes

Main trends in meal frequency and food served across the five children's homes in Kampala									
Children’s homes:		*Weekdays*				*Weekends/School holidays*			
		Breakfast	Lunch	Supper	Other meals	Breakfast	Lunch	Supper	Other meals
CH A	Girls aged 12-14 years	No	No	Posho and beans	No	No	No	Posho and beans	No
	Girls aged 15 -17 years	No	Posho and beans	Posho and beans	No	No	No	Posho and beans	No
CH B	Girls aged 12-14 years	Maize porridge with bread/doughnut	Posho and beans	Yes – menu changes	No – but they can get food when hungry	Maize porridge with bread/doughnut	Yes – menu changes	Meat/ chicken	No – but they can get food when hungry
	Girls aged 15-17 years	Maize porridge with bread/doughnut	Posho and beans	Yes – menu changes	No – but they can get food when hungry	Maize porridge with bread/doughnut	Yes – menu changes	Meat/ chicken	No – but they can get food when hungry
CH C	Girls aged 12-14 years	Porridge	Posho and beans	Posho and beans	No	No	No	Posho and beans	No
	Girls aged 15-17 years	No	Posho and beans	Posho and beans	No	No	No	Posho and beans	No
CH D	Girls aged 12-14 years	Maize porridge with bread/doughnut/egg	Yes – menu changes	Yes – menu changes	Yes – fruits and midday snack	Maize porridge with bread/doughnut/egg	Yes – menu changes	Meat/ chicken	Yes – fruits and midday snack
	Girls aged 15-17 years	Maize porridge with bread/doughnut/egg	Yes – menu changes	Yes – menu changes	Yes – fruits and midday snack	Maize porridge with bread/doughnut/egg	Yes – menu changes	Meat/ chicken	Yes – fruits and midday snack
CH E	Girls aged 12-14 years	Maize porridge	Posho and beans	Posho and beans	No	Porridge	Posho and beans	Posho and beans	No
	Girls aged 15-17 years	Maize porridge	Posho and beans	Posho and beans	No	Porridge	Posho and beans	Posho and beans	No

### Participants’ experiences with food and eating patterns

The two groups of participants at CH A had different opinions regarding satisfactions with food and eating patterns. The youngest girls, aged 12 – 14 years, who only ate one meal of posho and beans a day, expressed much more dissatisfaction regarding food and eating patterns compared to the girls aged 15 – 17 years. The former group was generally dissatisfied with the food, and there was a general consensus within this group that they were “sick of” eating the same meal every day:

*We eat for the sake of eating, cause it’s nothing to do, we just eat to live. But you feel even you don’t want to look at the posho.*

All participants in the youngest group discussed how they were very unhappy with the meal frequency. They used the word “hunger” and “hungry” many times, and explained how eating only one meal per day affects their lives:

*You can take some time, the whole day from morning to evening without taking (eating) any food, feeling very hungry. When you feel that the hunger is killing you! You think you will pass out from the hunger!*

The participants in the group aged 15 – 17 years, who ate twice a day as they received lunch at their school, said they were happy with their meal frequency:

*And even the food we are eating, because for us it is a great opportunity, it’s not a problem that we’re eating all posho and beans. Because that is what we have. But they give us enough food.*

When asked if they ever requested their caretakers for more food or go for second portions, all said they would never do that because their second portion might deprive another girl of food. They expressed that they understood that the home in general had a limited amount of food due to lack of financial resources, and that they would “certainly not” ask for more food than what had been put on their plate.

All participants in CH B, regardless of participating in the younger or older focus group, said that they were very content with the food and eating patterns. They also highlighted the quality of the food they ate, and said that the taste and freshness of the food was their biggest satisfaction. However, these girls had not always been that impressed with the food situation at the children’s home:

*They used to give us the same food as the ones they give the little kids. And then we said Eh! Those foods can’t satisfy us, so you add us, then they started mingling much more posho. The portion sizes, it was so small. Small portions. Then we complained, and they increased.*

Similar to CH A, the girls from CH C were also generally dissatisfied with the food and eating patterns including the meal frequency. All participants agreed that they did not like the food served to them, and everyone was tired of constantly eating posho and beans. The participants said that if they complained they would be chased away by the caretakers:

*They can even abuse you if you ask for more food! They can even beat you with your plate. And then tell you that you can’t have any more food.*

All girls said that if they could propose any changes, it would be to eat breakfast, lunch and supper, including evening tea before they go to sleep. Even though all participants expressed dissatisfaction regarding food and meal frequency, the girls aged 15 – 17 years expressed the most frustration over this issue. They said that they were discriminated against when it came to food and portion sizes.

All girls at CH D said that they were very content with the food and eating habits. They enjoyed eating a variety of foods and were satisfied with both the meal frequency and portion sizes. They further explained how they could decide their own portion sizes and always ask their caretakers for more food if they were not fully satisfied. They also emphasized their satisfaction towards eating different food almost every day:

*The food has so many different nutrients because it’s always different. For example fruits, we have them every day after supper. And the nutrients make us look good and our bodies look nice. I used to be so small and looking bad. Now I put on weight and feel energy.*

Most participants said that they like the food served to them at CH E because the quality of the food usually was of high standard, and even though they ate posho and beans every day, they liked it because it tasted good and were served in large quantities:

*They always fry the beans for us and the posho is always well cooked. Although I’m eating posho and beans throughout the week, it makes me happy. They only want the best for us here and make the best quality of the food that is available.*

However, some girls did have some dissatisfaction regarding the food:

*Sometimes you have a week, a whole week with beans that are not fried! You can even be scared and be thinking how am I going to take this food.*

There were some contradictory opinions regarding their satisfaction towards meal frequency. The youngest participants said they were comfortable with it. In contrast, the older girls highlighted the fact that they had experienced feeling hungry over a long period of time while living at the children’s home. For example on the day of the interview they had not eaten breakfast.

### Experiences with factors contributing to prevention and treatment of disease

CH A was only equipped with tap water. The girls explained that the lack of firewood to boil water is the biggest constraint in receiving clean drinking water:

*Sometimes they boil, because this year there are many young children here, they are not strong enough. Now they can’t take that boiled water, but for us, we are grown up and can handle it very well. But we know that it’s better to not drink from the tap.*

They could easily access health care when sickness occurred. CH A was equipped with one squat toilet to share. The availability of toilet paper was only randomly provided, and soap for washing hands was missing. Furthermore, the girls had difficulties accessing sanitary napkins:

*It is no fun being without those pads. It’s shaming, really. We use our clothes, old clothes that we have cut in pieces. Then we just wash them after use.*

The participants in CH B could access clean drinking water at all times. All residents had free access to toilet paper, sanitary napkins and soap. The girls shared one toilet. They were very satisfied with the availability of health services, and they also had a qualified nurse who lived with them permanently:

*They always have the best treatment for us and take it very seriously when someone is sick.*

The girls in CH C explained that they had access to clean drinking water on rare occasions. Participants from both the younger and older focus group pointed out other situations where they had feared to ask their caretakers about certain necessities. The staff could act abusive towards them if they asked. There was no availability of sanitary napkins, toilet paper or soap. CH C had nine toilets, some were squat toilets, others were flushing seat toilets but with water pipes collapsed. The younger girls said that they easily could access health care if they felt sick. The older girls had different experiences and said that the caretakers might discriminate them in providing health care:

*I don’t know when I’m going to the clinic, I haven’t told the caretaker yet (…) I didn’t tell her yet because she will just say: I don’t have money for medicine for you girls.*

All residents in CH D could always access clean drinking water, and they had always access to sanitary napkins, toilet paper and soap:

*Whenever something gets finished here, soap, pads, whatever, you just go and you request the matron for it and she gives you.*

CH D had two flushing toilets and many squat toilets, including two showers. Moreover, the girls highlighted that they were very happy with how this children’s home and its staff took care of them if they fell sick. They received the medication they needed and were taken to hospital when the sickness was of serious matter. A qualified nurse worked at the children’s home full-time. One girl told she was HIV-positive and that the caretakers made sure that she took antiretroviral drugs every day.

All residents in CH E had access to clean water. The girls had four showers and three toilets. Toilet paper and soap were always available. The two focus groups did, however, have different opinions regarding the availability of sanitary napkins. The girls aged 12 – 14 years said that the caretakers had bought boxes of sanitary napkins and that they bought more when stocks were empty. The older participants said that the caretakers used to provide them with sanitary napkins, but not anymore:

*The problem is money. So these days we are supposed to bring pads from our (previous) homes.*

The participants could access health care if they got sick and were very impressed about the treatment.

### Experiences with other care practices

All girls at CH A said they had a good relationship with their caretakers:

*These caretakers, if you have a good relationship with them they can show you true love, more than where I came from. They are like your parents. Every problem they are there for you. When you get problem and you talk to them, they help you.*

Many said that the caretakers treated them as if they were their own children and that the one-to-one relationship they had with an adult was good.

The girls at CH B also said they had a good relationship with their caretakers. They especially highlighted positive factors regarding communication. All residents and staff at the children’s home organised a weekly general meeting where they raised issues that concerned them and where every child could make complaints regarding the caretakers, food, arguments they had had with other residents, etc. These meetings were reportedly open and honest and the girls did not fear saying what was on their mind:

*Even if they are big people, you don’t fear. Even if these uncles have done something wrong to you, you speak it there.*

The younger and older focus group participants in CH C had some different views regarding their relationship with the caretakers. Whereas the younger girls had a good relationship with the matron, the girls aged 15 – 17 years portrayed a very different picture of her. They said that her behaviour towards them could be bad:

*She doesn’t want us here. She did not appreciate our coming here. She complains about us. She doesn’t want us here. That’s why she doesn’t want us to have food. That’s why on every small thing she provokes us.*

All girls at CH D said they had a very good relationship with their caretakers. Each girl was allocated to a specific staff member who had the primary responsibility of that girl, and they met weekly for “open heart sessions” where they privately could discuss problems. All children said that they liked this one-to-one contact they had with an adult.

The girls aged 12 – 14 years at CH E said that the relationship with their caretakers was “good” and “okay”. They did not elaborate much on the subject, but clearly said that they went to their caretakers if they had the need to talk to someone. The girls in the older focus group also said that the relationship was okay, but added on some information:

*The woman is powerful! For me, I fear her!” “She is not tough, but if you annoy her, she can get tempered.*

No specific caretaker had the main responsibility for one girl. When asked if they thought the one-to-one connection they had with the caretakers was sufficient, all girls in the youngest group said “yes”. The older girls also said it was “okay”, and elaborated further:

*If you don’t… if you are living with somebody in the house, even if she’s obviously so hard to you (…) you make the possible way to be good to her because sometimes if you are not related, it’s a problem to you.*

## Discussion

### Food, health and care conditions in light of human rights standards and principles

Under international human rights law to which the Ugandan government adheres, and also through norms under constitutional law, all Ugandan children have the human rights to adequate food, to the highest attainable standard of physical and mental health, and to the right to active caring practices that go beyond protection. As adequate food, health and care are the necessary underlying conditions for good nutrition, it can be said that when fulfilled, each of these conditions implies the realisation of each of these rights, to combine in a human right to good nutrition. Thereby the normative conceptual framework for what constitutes good nutrition is adapted to depict attainment of human rights standards for food, health and care. The standards can be given further content by drawing on the interpretations in the GC to the respective international legal conventions in question here ICESCR and CRC, reflecting the official UN understanding of each legal provision through their convention committees or treaty bodies.

Based on the findings from the focus groups on the adolescent girls’ own experiences, attitudes and views regarding their own nutrition situation as reflected through food, health and care conditions in their respective children’s homes, the following considers the degree to which the conditions comply with standards and principles for the human rights to adequate food, health and care.

### Food conditions in light of standards for realisation of the right to adequate food

According to GC 12 to the Covenant on Economic, Social and Cultural Rights, article 11 which includes the right to adequate food, this right is realised when food is

- Nutritionally adequate in quality and quantity to fulfil individual dietary needs
- Safe to eat
- Culturally acceptable
- Physically and economically available and accessible on a stable basis

#### Nutritional adequacy in the children’s homes

With reference to Fig. 1, the only foods consumed at CH A, C and E were posho, beans and maize porridge. Such clearly unbalanced diets can cause both micro- and macronutrient deficiencies that can result in poor health and productivity. Although the full quality of the diet and proportion of undernourished girls residing at children’s homes is not examined in this thesis, the diet in CH A, C and E are obviously not adequate to meet individual dietary needs. The foods served at CH B and D had more variety, and the residents ate differently every day.

#### Non-nutrient based values of food

The younger participants at CH A and all participants at CH C expressed huge dissatisfaction towards the meal frequency and over their monotonous diet of posho and beans. They described horrible situations over feeling hunger over a long period of time, and explained how the lack of food also affects their ability to concentrate at school.

The GC12 defines the aspect of *cultural acceptability* as “the need also to take into account, as far as possible, *perceived non-nutrient-based values* attached to food and food consumption” [32]. GC12 does not elaborate further what non-nutrient based value of food and food consumption constitutes, but within its definition of cultural acceptability is an implication that food constitutes more than nutrients. For food to meet criteria of *adequacy,* it must not only be of nutritional value, but also meet other satisfactions *beyond* nutritional adequacy. In our understanding of point 11 of GC 12, enjoyment of the right to adequate food encompasses rights of eating food that meet requirements of adequacy in terms of satisfaction both with taste of the food and with meal frequency. The experiences of the younger residents at CH A and all residents at CH C demonstrate how non-nutrient values, such as satisfaction with food and eating patterns are not attended to by the management/staff.

The right to adequate food entails being free from hunger. The younger residents at CH A and all residents at CH C explained how daily suffering of hunger, eating food they did not like and not being provided with enough food – either in quality or quantity – not only breaches with their right to be free from hunger, but also breaches with these girls’ inherent human dignity.

#### Can the right to adequate food and freedom from hunger be said to be met by participants in the five children’s homes assessed in this study?

For all participants at CH A, C and E, one or more of the criteria for full realisation of the right to adequate food were not met. Hence, the full realisation of the right to adequate food and freedom from hunger is not realised. Even though some participants at these three homes feel that one or more criteria of adequate food is met, the right to adequate food is not realised by the residents at these homes: when one criteria is not met, the right to food and freedom of hunger is not fully realised.

The right to adequate food and freedom from hunger is not realised for participants at CH A, C and E. Determining that these rights were realised in CH B and D is, however, not possible as an assessment of two criteria for the human right to adequate food not has been assessed in this study: (i) a detailed assessment of the girls’ food intake – and hence an evaluation of whether or not food intake meets dietary individual needs was not made, and (ii) the degree to which the food complies to standards with food safety.

### Health conditions in light of standards for realisation of the right to health

The right to health does not mean the right to be healthy. However, desirable living conditions must be in place in order to protect oneself against potential sickness. According to GC 14 on the right to the highest attainable health, the right to health must be understood as a right to enjoy a variety of facilities, goods, services and conditions necessary for the realisation of the highest attainable standard of health [33].

#### Access to health care

The older participants at CH C were the only ones who said they do not access health care when sickness occurs. They explained that the caretakers were not bothered about their illnesses, and had chased them away when they told they were not feeling well. The right to appropriate healthcare is thus not realised for these participants since their needs of treatment was neglected. All other participants in this study said that they easily can access healthcare when sickness occurs.

#### Access to basic necessities

The unhygienic situations in CH A and C did not promote conditions that are necessary for the highest attainable standard of health. On the contrary, the complete lack of soap, toilet paper and, in the case of CH C residents, ample lack of water for any kind of use - put these girls in huge risk of developing food- and water borne diseases as well as diarrhoeal diseases. The residents are, however, entitled to these necessities under international human rights law, and the fact that they are not, breaches with their enjoyment of the right to the highest attainable standard of health. None of the residents at CH A or CH C have stable access to clean drinking water either, which is a violation on their right to water [35].

The residents at CH B, D and E had a constant availability of clean drinking water. They were also content with how their caretakers handled situations when sickness occurred. Furthermore, the girls were provided with toilet paper, running water and soap for hand washing, which are vital necessities in fighting potential illness. It seems, based on the participants’ experiences, that the measures CH C, B and E take to promote health through providing the above mentioned necessities is taken well care of by the management. However, since we have too limited data to determine if some residents enjoy the full realisation of the right to adequate food, the same applies in terms of determining if the right to the highest attainable standard of physical and mental health is realised for the residents in CH B, D and E, as an assessment of all facilities necessary for realisation of the right to health is lacking.

GC 14 does not pronounce which specific necessities and facilities right-holders are entitled to. Consequently, assessing if the right to the highest attainable standard of health is realised for anyone, is particularly challenging given the vaguely spelled-out language in GC 14.

### Care conditions in light of standards for the right to care and the right of the child to be heard

Although there is no GC on the right to care, the CRC does explicitly recognise the right to care of children. The right has been interpreted by Jonsson as active caring practices that go beyond protection [36]. But what constitute “care beyond protection” and “active caring practices”? That definition by the ICN in 1992 [15] can be contextualized to children’s homes as follows: “time, attention and support of caretakers to meet physical, mental and social needs of residents”.

Active caring practices beyond protection can as such be seen as use of time, attention and support of caretakers. Caretakers must have adequate *time* for residents and include listening to them and taking time to hear them, i.e. putting in more efforts than simply acknowledging their presence. *Attention* can relate to *responsiveness*– that the views of residents are responded to, i.e. that their opinions and views are taken into account and are taken seriously by caretakers. *Support* can relate to practices that enhance physical, mental and social needs of residents, and may thus include warm and sensitive caregiver-child interactions. Furthermore, support can constitute a combination of time and attention, or more precisely, the result of adequate time and adequate attention. In sum, if caretakers take the time to see children as active, participatory human beings, by letting them express their views freely and taking them seriously through paying attention to their views, residents are then more likely to be actively supported. On this basis, active caring practices and care in children’s homes correspond with GC12 of the CRC which recognises the rights of children to be heard, freely express opinions and be taken seriously [34].

The results from this study confirm how the aspect of care is of paramount importance in terms of safeguarding nutrition security. The results also show how realisation of the right of a child to be heard (express oneself and be taken seriously) affects realisation of other rights as well. At CH C, residents are “chased away” when they ask for more food, and hear that they are not worthy because they are “orphans from poverty”. At CH C, the fact that caretakers do not attend to the rights of children to be heard, consequently suppresses the residents’ realisation of the right to adequate food. The caretakers’ unwillingness to listen to the residents puts them in a situation where they are incapable of claiming their rights as rights-holders.

Other examples from CH C also demonstrate how inadequate caring practices infringe with the residents’ realisation of the right to the highest attainable standard of physical and mental health: the older residents pointed out that the caretaker can deny them medical treatment when sickness occurs. Another girl said she feared she had malaria, but had not yet told the matron in fear of a negative response. In contrast, residents at CH B and D were encouraged to freely express their opinions, as these homes arrange weekly scheduled meetings with all residents and staff members. The participants at CH B and D did not fear complaining about any matters in the children’s home context, and if they did, their complaints were well-received and changes were made accordingly by the staff. Residents at CH B and D said that their complaints with food quality and quantity resulted in a more balanced diet. This exemplifies how the rights of children to be heard by and large correspond with a progressive realisation of other rights, in the example from CH B and D, the right to adequate food.

As explained by residents at CH A and E, there is reason to believe that financial constraints and lack of resources limit the residents’ realisation of the human rights to adequate food and health. In contrast, at CH C lack of adequate care also seemed to be a dominant factor for the suppression of realisation of the resident’s rights.

These examples support existing recommendations of how care is a significant determinant of nutrition security. Although the existing recommendations highlights the care aspect in terms of ensuring nutritional adequacy especially for young children [16, 19, 37], the results from this study suggest that care in the children’s homes is relevant for nutrition security also of the adolescent girls participating in this study. This particular finding may be relevant in other children’s homes as well or in general in residential care facilities where residents are dependent on provision of food and healthcare by caretakers.

### The findings in light of human rights principles

Respecting and applying the principles of a HRBA can enhance the creation of an enabling environment where human rights are more likely to be realised. Analysing the findings in light of human rights principles can contribute to an understanding of why – or why not – the conditions in children’s homes complied with specific standards for the rights to adequate food, health and care.

#### Participation and empowerment

Only when children are taken seriously and seen as active human beings whose opinions matter, they can truly participate and contribute in decisions that affect their lives. Article 12 of the CRC on the rights of children to be heard can be interpreted as rights of children to be empowered through participation. Our findings demonstrated how degrees of participation and empowerment correlated to whether or not standards for realisation of the right to food were met. At CH C, residents were chased away when they asked for more food, and heard that they are not worthy because they were “orphans from poverty”. The caretakers’ unwillingness to listen put the residents in a situation where they were incapable of claiming their rights as rights-holders.

In contrast, residents at CH B and D were encouraged to freely express their opinions. The participants highlighted the fact that they did not fear complaining about any matters in the children’s home context, and if they did, their complaints were well-received and changes were made accordingly by the staff. Residents at CH B and D said that their complaints with food quality and quantity resulted in a more balanced diet. This exemplifies how high degrees of participation and empowerment by and large correspond with a progressive realisation of other rights, such as the right to adequate food.

#### Non-discrimination

It became clear that participants at CH C had experienced a discriminatory behaviour from the caretakers. The residents explained how the caretakers had made them feel unworthy of food because they were “orphans from poverty”. The residents also shared stories on how the caretakers would abuse them if they asked for more food. The older girls further said that the caretakers discriminated them according to their age. The principle of non-discrimination takes roots in how every human is born equal, and are entitled to the same rights as any others. The stories described by CH C-residents portrayed a picture of how discrimination is present even in a children’s home, a setting where some of the most vulnerable members of society live. The older participants at that home also explained how the discrimination towards them infringed with their realisation of the right to food, health and care. In CH B and D, however, non-discrimination was promoted as children and caretakers are “equalized” in the sense that they all met to discuss potential complaints, opinions and changes within the children’s home setting.

#### Human dignity

When human rights are ignored, human dignity is not attended to. The participants’ experiences with suffering from hunger, eating food they did not like on a daily basis, lack of water, soap, toilet paper and sanitary napkins, are all examples of undignified situations. Although the lack of such necessities infringes with the girls’ dignity, the lack of financial resources seemed to be the major cause of this. It was only the participants at CH C who described situations where it was the caretakers’ behaviour towards them that infringed with their inherent dignity, and exemplifies how the caretakers themselves were the source of the problem.

#### Respect for the rule of law; accountability

As regards the human rights principles *respect for the rule of law* and *accountability*, the conditions in the homes can be compared with the requirements set by the MGLSD in these regulations for children’s homes [12]. Some examples of cull compliance: all homes complied with regulation 8(4): *The home shall be run in such as manner that there should be an officer on duty at the home at all times*, and furthermore with regulation 13: *Each home shall ensure appropriate education to each child.*

Regulation 8(6) proclaims: *Care in each home shall be on a twenty-four hour basis.* All children’s’ homes had caretakers living at the children’s homes with them, however, care was not always provided at children’s homes, especially not at CH C. It is therefore not appropriate to establish that “care” was available on a 24-h basis in all homes, even though a caretaker was present 24 h of the day. Regulation 8 (5) proclaims that caretaker/child ratio in homes should be 1:8. Only CH B complied with this regulation. Furthermore, only CH B and D had a registered nurse who works at the children’s home full time (regulation 9 (1)). Most astonishing, however, is that none of the five homes comply with regulation 3 (1) which proclaims that: *no home shall operate unless the homes have been approved in writing by the minister*. This means, in principle, that none of the homes are allowed to operate as children’s homes.

### Our findings in light of other studies

Besides of the results from this study, there is not much knowledge regarding food and eating patterns at children’s homes in Uganda in general. The only existing data are the results of MGLSD’s study in 2011, which revealed that diet in children’s homes was limited [13]. These findings are consistent with the findings from CH A, C and E. Studies conducted in Bangladesh and India found that on average three meals per day are served in similar institutions [38, 39]. Sadik assessed food consumption patterns and dietary diversity in an orphanage in Ghana, and concluded that the residents’ diet was low both in micro- and macronutrients [40]. These findings seem comparable to the eating patterns in CH A, C and E. Ribeira et al. compared two child care institutions in Ghana, and found on the other hand that residents at each home received adequate quantities of vegetables, fruits, legumes, meat, eggs and fat-rich foods [41]. Shanbhog et al. investigated five children’s homes across India. Although they did not elaborate much around the details of the diet, they found that the residents were provided with “nutritious, healthy and fibrous foods” [42]. The children’s homes in these latter two studies may be more comparable to the food intake of residents in CH B and D.

According to *Save the Children*, provision of food and other necessities in child care institutions may vary across different countries [43]. Adding to this, the findings from this study reveal that differences in children’s homes occur not only in the same country, but also across children’s homes in the same city and even between children living in the same institution.

*The Comprehensive Food Security and Vulnerability Analysis* demonstrated that poor food consumption affects nearly 5 % of the Ugandan population, and thus the residents at CH A, C and E might be worse off than 95 % of the rest of the population [44].

While this study indicates that some children’s homes residents experience more food insecurity compared to the rest of the population, other researchers have in fact found a protective effect of institutionalization in terms of food intake. Braitstein et al. did a comparison of physical and mental health of children who were either living in children’s homes or in regular households in Western Kenya [45]. They found that institutionalized children were more food secure and had better nutritional status compared with children living in regular households. Panpanich et al. also found that children living in institutions have greater long-term food security compared to their age- and sex counterparts in other living arrangements [46].

All participants, except the older girls at CH C, said they had access to health care when sickness occurred. Residents at CH A and C were in complete lack of soap, toilet paper, sanitary napkins, clean drinking water whereas residents at CH B, D, and E had stable access to such necessities. Data from the Uganda Demographic and Health Survey (UDHS) reveal that only 27 % of Ugandan households have access to running water and soap [7]. Results from our study showed that in 60 % of the children’s homes, access to soap was adequate. However, due to the small sample size in this study we cannot conclude that children’s homes residents in general are more likely to have soap- and water access compared to other Ugandan households. The MGLSD found in their study that access to clean drinking water in the 40 investigated children’s homes was scarce. These findings are consistent with the findings from CH A and C. Moreover, data from UDHS revealed that 56 % of Ugandan households lack access to clean drinking water, demonstrating that the findings from these two children’s homes were rather similar to the rest of the nation [7].

Most participants portrayed their relationship with caretakers as good. The exception was the older residents at CH C. Studies suggest that many children in child care institutions are likely to suffer from mental health problems and development delays [47–49]. The residents’ mental health status was not examined in our study. The St. Petersburg-USA Orphanage Research Team and Rutter have, however, found that the leading causes of emotional distress experienced by institutionalized children is the lack of adequate caring practices, where warm, emotional interactions between caretakers and residents is absent [50, 51]. The lack of such adequate caring practices of caretakers might be comparable to the situation of residents in CH C. McCall et al. conducted an inventory of caretakers’ behaviour in children’s homes in Russia and found that training staff to approach children with warm, sensitive and responsive caregiver-child interactions significantly correlated with better developmental scores of the children [52].

### Limitations of the study

To some degree, the exclusive focus on adolescent residents’ experiences is a limitation to this study. Including caretakers’ perspectives on food, health and care conditions in the children’s homes would have allowed a validation and evaluation of two different perspectives of the same phenomena, and for a more comprehensive picture. Furthermore, inclusion of more children’s homes in Kampala might have expanded our knowledge on how food, health and care conditions are experienced by children’s homes residents.

The food intake and dietary diversity were not assessed in depth and consequently the nutritional adequacy of the diet could not be determined. For studies using qualitative methods it is impossible to make generalisations of the findings to a given population, due to small, non-geographically representative sample size [25], such as the present study. Finally, future studies should include adolescent boys’ perspective as well, as they may have different, specific needs that must be attended to.

## Conclusions

To our knowledge, this study is the first to consider food, health and care conditions in children’s homes in relation to the standards of food, health and care as human rights. Food, health and care conditions as experienced by the adolescent girls varied greatly across the five children’s homes. By comparing conditions of food, health and care, as experienced by female adolescent residents, to provisions in specific general comments, we found that:

- One or more standards for the right to adequate food were not met at children’s homes A, C and E, and thus, the full realisation of the right to adequate food cannot be said to be met by the residents at these homes.
- One or more standards for the right to the highest attainable standard of physical and mental health were not met at children’s homes A and C, and thus, the full realisation of the right to health cannot be said to be met by the residents at these homes.
- The full realisation of the right to care (interpreted through, *inter alia,* the right of the child to be heard) cannot be said to be met by the residents at children’s home C.
- The data in this study are too limited to indicate that these rights were realised in homes where the residents described their conditions as good.
- Care in the children’s homes was a contributing factor for whether or not standards for the rights to adequate food and health are met.

All duty-bearers with obligations and responsibilities towards realisation of rights in children’s homes should, within the maximum extent of their resources, uphold their obligations for this particularly vulnerable group constituted by female adolescent children’s home residents. There is obviously no question that all children’s homes in Uganda should provide their residents with a variety of foods that comply with the standards enshrined in GC 12 (ICESCR) on the right to adequate food, facilitate health promoting environments that allows for a realisation of the right to the highest attainable standard of health, and provide active caring practices that goes beyond protection and comply with standards of GC12 (CRC) on the right of the child to be heard.

## Abbreviations

CESCR: Committee on Economic, Social and Cultural Rights
CRC: Convention on the Rights of the Child
GC: General comment
GOU: Government of Uganda
HRBA: human rights-based approach
ICESCR: International Covenant on Economic, Social and Cultural Rights
ICN: International Conference on Nutrition
MGLSD: Ministry of Gender, Labour and Social Development
STC: systematic text condensation
